# High‐Intensity Gait Training for Patients After Stroke: A Feasibility Study

**DOI:** 10.1002/pri.70059

**Published:** 2025-04-10

**Authors:** Iris Charlotte Brunner, Gunhild Mo Hansen

**Affiliations:** ^1^ Hammel Neurocenter/Aarhus University Hammel Denmark; ^2^ Department of Physiotherapy University College of Northern Denmark Aalborg Denmark

**Keywords:** gait training, physiotherapy, rehabilitation, stroke

## Abstract

**Background and Purpose:**

Approximately two‐thirds of stroke patients experience various levels of walking impairment that limit their participation in society. Mounting evidence suggests that gait training provided at high cardiovascular intensity with a focus on stepping practice improves gait function after stroke and is superior to lower intensity standard gait training. However, high intensity gait training (HIGT) is not widely applied.

**Purpose:**

With this study, we wanted to examine the feasibility of HIGT in a XXX neurorehabilitation hospital.

**Methods:**

A longitudinal cohort study with 15 patients participated in 2 weeks of HIGT with 3‐5 sessions per week. HIGT was provided as part of standard physical therapy. The results included feasibility measures such as adherence and fidelity to treatment, adverse events, and patient satisfaction. Furthermore, gait assessments were performed before and after the intervention and heart rate and number of steps were monitored during the training sessions.

**Results:**

Eleven of the 15 patients were non‐ambulatory or dependent on the support of two people at the start of HIGT. Adherence to treatment was good, with almost all (14/15) completing 8 sessions or more. No serious adverse events occurred. The target heart rate of > 60% of HR_max_ was achieved for a mean of 26.4, SD 7.4, min‐max 12.3–37.0 min per session. The number of steps increased from 245.44 (SD 223.12) in the first session to 676.75 (SD 376.83) in the last session. However, with a large variety, both within and between individuals. There was a significant improvement in all gait assessments. Patient satisfaction was high.

**Discussion:**

HIGT was feasible, well tolerated by the patients and could be provided within existing staffing levels. There were no serious adverse events, and all patients confirmed that they would recommend HIGT to a friend in the same situation.

## 1 Introduction

Regaining walking ability is one of the priorities for stroke patients (Preston et al. 2021). Approximately two‐thirds of stroke patients experience various levels of walking impairment (Preston et al. 2021). Although many regain some walking ability, their gait is still impaired in terms of reduced speed, range, and safety (Pollock et al. 2011).

A substantial proportion, 40% of initially nonambulatory patients are not able to walk independently 3 months after stroke (Preston et al. 2021). Physical functioning and independence in daily activities are closely related to quality of life, caregiver burden, and health care utilization (Wohlrab et al. 2022; van Mierlo et al. 2016). Furthermore, reduced mobility limits participation in valued social activities (Ramos‐Lima et al. 2018) and increases the risk of recurrent strokes and other adverse outcomes (Kang et al. 2021).

Research suggests that gait training provided at high cardiovascular intensity with a focus on stepping practice improves gait function after stroke and is superior to lower intensity standard gait training (Boyne et al. 2023; Hornby, Holleran, Hennessy, Leddy, Connolly, Camardo, Woodward, Mahtani, Lovell, and Roth 2016a; Klassen et al. 2020; J. L. Moore et al. 2020). Previous research found that the amount of practice during an average physiotherapy session in different rehabilitation settings was as low as 357 steps and as few as 288 repetitions (Lang et al. 2009; Scrivener et al. 2012). The authors of a recent Cochrane review on physical fitness training for stroke patients recommended cardiorespiratory training, including walking (Saunders et al. 2020). The effectiveness of gait training at high cardiovascular intensity (HIGT) is also supported by a recent meta‐analysis by Tapp et al. (Tapp et al. 2024 jul). HIGT combines several factors that are crucial for motor recovery after stroke, namely specific, repetitive, intensive, and salient training (Kleim and Jones 2008).

Although scientific evidence supports HIGT and its application as recommended in the Canadian Stroke Best Practice Recommendations, the US American Guidelines for Stroke Rehabilitation, and the Clinical Practice Guideline to Improve Locomotor Function Following Chronic Stroke, Incomplete Spinal Cord Injury, and Brain Injury (Teasell et al. 2020; Winstein et al. 2016; Hornby et al. 2020 jan), it is not widely implemented. The time lag for knowledge transfer from research to clinical is substantial and structured efforts are needed to diminish it (Walker et al. 2013). In most cases, adaptations to a specific context are needed to ensure that the intervention can be applied appropriately (G. Moore et al. 2021).

This study was a preparatory step of an implementation process in which we wanted to investigate the feasibility of HIGT in a XXX neurorehabilitation hospital. Different aspects of feasibility, such as adherence (numbers of sessions and heart rate and steps recorded), fidelity (minutes spent > 60 HRmax), acceptability of HIGT for both patients and therapists, and tolerability (adverse events, self‐reported experience), were examined.

## 2 Methods

### 2.1 Design and Setting

We conducted a longitudinal cohort study with test sessions prior and post‐HIGT. All patients were admitted to two wards at the XXX Neurorehabilitation Center in the period of 1. September 2022 to 1. March 2023 were selected for eligibility by a trained HIGT physiotherapist and evaluated by a physician to rule out any risk factors, such as atrial fibrillation or hypertension that is difficult to regulate. Patients aged 18 years or older who had experienced a stroke were included in the study, provided they were capable of giving informed consent and could comply with HIGT. The determination of a patient's ability to comply with HIGT was made by the treating healthcare team, primarily based on an assessment of the patient's cognitive capacity to understand the requirements of HIGT. Additionally, the patient needed to have a primary rehabilitation goal of improving gait function. Patients were excluded if they suffered from uncontrolled cardiovascular, metabolic, or psychiatric problems, were in need of equipment that limits gait (e.g., ventilator), or could not walk with or without walking aid > 50 m before their current hospital stay.

### 2.2 The Intervention

Physiotherapists (PT) Who had Attended Specific Training in HIGT Provided the Intervention. The Core Components of HIGT

- Focus on stepping practice with challenging and varied repetitions. PTs encouraged patients to perform as much stepping practice as possible during a training session. Stepping practice included many variations, such as walking indoors, outdoors, on stairs, on the ground, on treadmills, with a gait robot, and weight support as needed. Moreover, walking was practiced forward, backward, sideways, and over obstacles as examined in other studies (J. L. Moore et al. 2020; Hornby, Holleran, Hennessy, Leddy, Connolly, Camardo, Woodward, Mahtani, Lovell, and Roth 2016b; Holleran et al. 2014).
- Targeted heart rate of 70%–85% of the maximum heart rate predicted (HR_max)_ based on the formula 208‐(age *x* 0.7), minus 15 if blood pressure is high, defined as ≥ 130/80 over several assessments (Tanaka et al. 2001 jan; Stafford 2018 oct). A target HR of 60%–70% could be applied for medical reasons according to the assessment of the physician responsible.
- Deprioritizing other activities during a PT session, such as balance training, transfers, and standing.

### 2.3 Feasibility Outcomes

Adherence was assessed in terms of the number of sessions conducted as intended, registrations of heart rate during sessions, and steps conducted during sessions. Fidelity was assessed based on how many minutes were spent in > 60 HRmax. Tolerability was assessed with the registration of adverse events. Acceptability of HIGT for patients was assessed with a customized questionnaire. The patients were asked to provide their opinion on the following statements.

1. My gait function improved after high intensity gait training.
2. High‐intensity gait training was well adapted.
3. High‐intensity gait training was too exhausting
4. It was easy to communicate with the therapists.
5. I received sufficient information on high intensity gait training.
6. My concerns were taken seriously
7. I would recommend high intensity gait training to a friend

Response alternatives were as follows: True, Partly true, Not true, or Don't know.

In an open‐ended question at the end, patients could write other comments.

We anticipated that at least 90% of responses for the positive statements (Preston et al. 2021; Pollock et al. 2011; van Mierlo et al. 2016; Ramos‐Lima et al. 2018; Kang et al. 2021; Kang et al. 2021; Boyne et al. 2023) would be categorized as either “True” or “Partly true.” Additionally, we expected that at least 90% of responses for the negative statement (Wohlrab et al. 2022) would be classified as "Not true" in order to ensure sufficient patient satisfaction.

How therapists experienced the introduction of HIGT was explored with qualitative interviews and in informal discussions (data not presented here).

### 2.4 Assessments

Physiotherapists trained in HIGT tested patients before and after HIGT. The clinical outcome measures were 10‐m walk test (10MWT) (van Bloemendaal et al. 2012), 6‐min walk test (6MWT) (Flansbjer, Holmback, Downham, Patten, & Lexell) (if patients were able to walk longer distances), Timed up and go (TUG) (Podsiadlo and Richardson 1991), Functional ambulation categories (FAC) (Lundquist and Brunner 2023), 30 s Sit‐to‐stand (Csuka and McCarty 1985). The BORG The Perceived Exertion Rate (RPE) was applied after the training sessions (Borg 1970).

### 2.5 Monitoring of Steps and Heart Rate

To count steps, patients wore a Modus StepWatch monitor (Modus Inc., Washington, DC) on the paretic ankle. StepWatch has been validated for research purposes (Fulk et al. 2014). Since most of the patients were unable to walk at the beginning of the intervention, step watches were only worn during training sessions.

Heart rates were monitored throughout the training sessions using the Polar Verity Sense device. The objective was to maximize the duration spent in an intense heart rate zone, ideally between 70% and 85% of HRmax, or at a minimum of over 60% of HRmax.

### 2.6 Data Analysis

The characteristics of the patients, the heart rate, and the step counts were displayed with descriptive statistics.

Likewise, feasibility outcomes such as the number of sessions were presented in absolute numbers, and percentage, as mean and SD, adverse events in absolute numbers. The number of minutes spent in > 60% of HR_max_ 70%–85% of HR_max_ was merged into one group and presented as mean (SD). Patient satisfaction was presented in absolute numbers for each category.

Due to the small sample size and the fact that the differences between pre‐ and post‐intervention assessments were not normally distributed Related samples, the Wilcoxon Signed Rank test was chosen for the analysis of per‐ and post‐intervention differences of gait assessments and the sit‐to‐stand test.

### 2.7 Ethics

All patients provided their written informed consent. The study was assessed as not required to receive approval from the XXX Ethics Committee.

## 3 Results/Findings

Fifteen patients were included, most of them with complex neurological challenges due to stroke, as the hospital in general treats patients with moderate to severe impairments. Four out of 15 patients were nonambulatory (FAC = 0) and seven were dependent on continuous physical support (FAC = 1) when starting HIGT, Table 1. Their functional independence measure (FIM) scores reflect a generally high need for support in daily activities with a mean of 66.3 (SD 25) of 126 possible for the entire FIM scale and a mean of 42.6 (SD 19.8) of 91 possible in the motor domain of the FIM scale, Table 2.

**TABLE 1:** Gait and strength assessments.

Assessment	Pre‐intervention	Post‐intervention	*p*‐value
Functional ambulation categories (median/min, max) (0‐5 best)	1 (0, 4)	2 (0, 5)	0.001
10 meter walk test, m/s median (min, max)	0.10 (0, 0.8)	0.44 (0, 1.47)	0.001
Sit‐to‐stand, repetitions median (min, max)	5 (0, 12)	8 (0, 13)	0.001
6 minute walk test, m median (min, max)	40 (0, 260)	91 (0, 391)	0.001

**TABLE 2:** Demographic and clinical characteristics, *N* = 15.

Age, years (SD)	55.6 (11.6)
Sex (male), *n* (%)	9 (60)
Time since stroke, *d* (SD)	44.3 (29.2)
Side of paresis right *n* (%)	9 (60)
Stroke severity NIHSS (SD)	24.6 (23.1)
NIHSS categories: Severe/moderate/mild, n	7/5/1
Ischemic/hemorrhagic/both	7/6/2
FIM at enrollment (19–126)	66.3 (25.0)
FIM motor scale at enrollment	42.6 (19.8)

### 3.1 The Feasibility Results

#### 3.1.1 Adherence to Treatment

We examined whether a minimum of eight sessions could be conducted and whether heart rate data and step counts could be collected as intended. Of the 15 patients, almost all, 14, could complete at least eight sessions, seven of those received nine, and five received 10 sessions. One patient completed only 4 sessions due to sick leave from the treating therapist. Some technical challenges, such as equipment not recording, difficulty connecting to phones or tablet PCs, or just not charging sufficiently, were encountered when collecting heart rate and step count data. Of the heart rate data, 13% were missing, and the step count data was missing for 22% of the sessions.

#### 3.1.2 Treatment Fidelity

**FIGURE 1:**
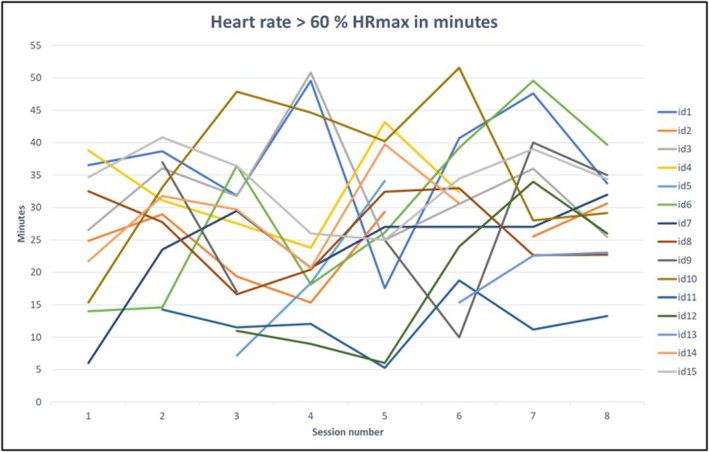
Time spent at a heart rate of > 60 HRmax for each session and patient. Both categories of > 60%‐70% and 70%‐85% were collapsed into one category > 60%.

**FIGURE 2:**
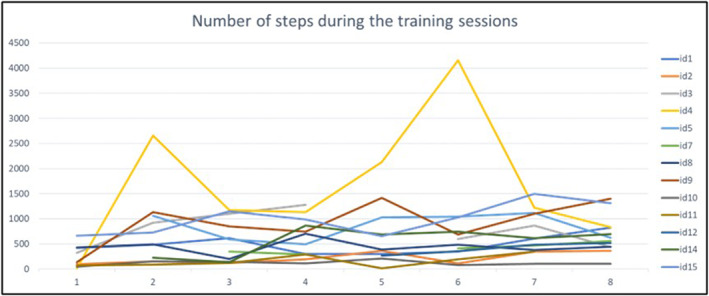
Number of steps during each session.

There was no defined minimum number of minutes in > 60% heart rate or number of steps to be achieved. We observed a large degree of variability both within and between individual patients, as shown in Figure 1 for heart rate and Figure 2 for step counts. The mean number of minutes spent in > 60% HRmax was 26.4, SD 7.4, min‐max 12.3–37.0, Figure 1. The number of steps increased from 245.44 (SD 223.12) in the first session to 676.75 (SD 376.83) in the last session. However, with a large variety, both within and between individuals, as can be inferred from Figure 2.

#### 3.1.3 Adverse Events

In general, HIGT was well tolerated, and no serious adverse events occurred. One patient reported pain in her knee that she had also experienced earlier due to arthritis. Pain did not prevent them from continuing HIGT.

#### 3.1.4 Satisfaction With HIGT

Of the 15 patients, 12 (80%) completed the patient satisfaction questionnaire. Three could not complete it due to aphasia and other cognitive problems. Eleven of those confirmed stated 'True' for improving gait function, one 'Partly true'. The same distribution was applied to the statements 'High‐intensity gait training was well adapted' and 'It was easy to communicate with the therapists', with 11 checking 'True' and 'Partly true'. Ten patients felt that their concerns were taken seriously, while two stated “Partly true”. One patient found HIGT to be too exhausting, while 11 did not. Ten said that they had been provided with sufficient information; for one person this was 'partly true' and one checked 'Don't know'. All agreed that they would recommend HIGT to a friend in the same situation.

### 3.2 Gait Function and Related Assessments

For FAC, the number of patients who were not ambulatory or dependent on two persons to walk (categories 0 and 1) at the start of the intervention decreased from 11 to 6. Nine out of 15 patients were unable to perform the TUG before the intervention, and six of them still faced challenges after the intervention. The six patients who could perform the TUG improved from median (min, max) 19.5 (5, 73) at baseline to 12.0 (9, 47) at post‐intervention. There was a significant improvement in all gait assessments from baseline to post‐intervention, Table 1.

## 4 Discussion

HIGT was feasible and well tolerated by the patients and there were no serious adverse events. The most important feasibility outcome for clinical practice, besides the absence of adverse events, was that HIGT could be integrated into clinical practice. This was reflected in the fact that most patients, apart from one, were provided with the planned minimum eight training sessions and several attended more than eight. HIGT could be realized within existing staffing levels, a crucial point for further implementation.

HIGT was very positively received by all patients who responded, confirming that they would recommend this approach to a friend in the same situation. However, our participants may have represented a selected group in that they were deemed suitable to participate by the neurorehabilitation team. They may also have been a very motivated group, since they had provided informed consent and were aware that they participated in a treatment program. However, earlier studies in Norway and the US have convincingly demonstrated that intensive stepping training could be implemented in hospital care for a broad range of patients with stroke (J. L. Moore et al. 2020; Henderson et al. 2022 sep).

Although data collection on the number of steps and heart rate was satisfactory, it could have been more complete. Of the heart rate data, 87% could be registered and 78% of the step counts. These data suggest that step monitoring provided more difficulties. Therapists reported connectivity issues and insufficiently charged monitors as the main reasons for missing data. This emphasizes the need for training of therapists and a well‐organized storage of step and heart rate monitors. Furthermore, there is still room for improvement in the user‐friendliness of monitors to facilitate use in clinical practice (Lang et al. 2020). In a busy clinical practice, the use of one device for heart rate and step count, and not one for each, would be more convenient.

Gait function improved in most patients. However, the observed improvement could also result from spontaneous biological recovery, which typically occurs in the early stages after a stroke, as well as from other forms of rehabilitation. Without a control group, it is impossible to isolate the specific effects of HIGT from those of other rehabilitation interventions. We were able to include many patients (11 of 15) with severe gait impairments, scores 0 and 1 on FAC, and a generally high level of dependence, as reflected in a FIM score of 66.3 (25.0) of maximal 126 at enrollment, and a more severe stroke as indicated by the mean NIHSS score of 24.6 (23.1) of 42 possible. Studies by Klassen et al. (Klassen et al. 2020) and Moore et al. (J. L. Moore et al. 2020) included patients who were on average less severely impaired. The patients in the first study had a mean NIHSS score (SD) of 5 (Wohlrab et al. 2022). The latter study does not report NIHSS or FIM scores, but a modified Rankin Scale score of 3.3 (0.87) also suggests that their patients were less affected. In a large study conducted by Henderson et al., patients within 2 months after stroke, who were more severely affected were included (Henderson et al. 2022 sep). Their outcomes were compared to historical samples from before the implementation of HIT (*n* = 131) and during a transition phase (*n* = 317). Those who participated in HIT exhibited greater improvements in gait and other outcomes. Interestingly, the study allowed for an 18‐month transition period for the implementation of HIT, highlighting the importance of fostering changes in local cultures and securing management support for successful implementation after initial feasibility testing.

Studies in which patients in the chronic phase were included required them to be ambulatory to some extent (Boyne et al. 2023; Podsiadlo and Richardson 1991). Therefore, the collective evidence indicates that HIGT is applicable to patients with a wide range of gait impairments. Though a recent study by Thomson et al. showed that high‐intensity walking alone did not increase the number of daily steps and behavioral interventions such as step monitoring may be necessary to increase activity in the chronic phase (Thompson et al. 2024 jan).

The inclusion of severely impaired patients may also explain the rather erratic development of minutes spent in > 60% HRmax and step counts. There was no linear development from the first to the last session. In many cases, this could be explained by the fact that the patients improved their gait function. When the assistance was reduced, the number of steps could decrease. The same applies to heart rate. Although it was relatively easy to increase heart rate gait training with a body weight supporting harness by increasing velocity, this could be less easily done when the person walked independently with a cane due to safety concerns. Moreover, there were other variations in fitness from day to day. The progression goals, such as the model developed by Peters et al. (Peters et al. 2022), provide useful guidelines for an ambulatory population but may be difficult to apply to severely impaired patients.

## 5 Limitations

The primary limitation of this study lies in the potentially selective inclusion of motivated patients. As this was the first attempt to implement HIGT at our hospital, practical considerations influenced patient selection. For instance, inclusion was partly based on the availability of therapists trained in HIGT at the ward. Additionally, the treating neurorehabilitation team assessed patients' ability to comply with the training, which may have introduced some selection bias. Despite this, the resulting sample appears representative of a significant portion of patients admitted to the hospital, including those with substantial gait and motor impairments as well as some cognitive challenges.

The absence of a control group limits the ability to attribute changes in gait function to the intervention specifically. Furthermore, the small sample size restricts the generalizability of the findings.

Another limitation was that the physiotherapists conducting the training had limited prior experience with HIGT and the technical equipment used for monitoring. Nevertheless, they had extensive expertise in neurorehabilitation.

## 6 Implications for Physiotherapy Practice

HIGT was highly feasible in a group that included many non‐ambulatory patients as well as those requiring the support of two people. The patients expressed high levels of satisfaction with the training. HIGT seems promising for a broad range of patients with gait impairments after stroke. Our results suggest that even patients with substantial gait challenges can benefit from HIGT.

## Author Contributions

Both authors contributed to the design of the study, data analysis and writing.

## Ethics Statement

The study was assessed as not required to receive approval from the Central Jutland Region Ethics Committee, case number 1–10‐72–21‐22.

## Consent

All patients provided their written informed consent.

## Conflicts of Interest

The authors declare no conflicts of interest.

## Data Availability

Data can be obtained from the first author upon reasonable request.
